# Diversity, characteristics, and abundance of native arbuscular mycorrhizal fungi in the semi-arid lands of Eastern Kenya

**DOI:** 10.3389/fmicb.2025.1582476

**Published:** 2025-05-21

**Authors:** Michael Sakha, Joseph P. Gweyi-Onyango, Cargele Masso, Frederick P. Baijukya

**Affiliations:** ^1^Department of Agricultural Science and Technology, Kenyatta University, Nairobi, Kenya; ^2^One CGIAR, Impact Area Platform on Environmental Health and Biodiversity, Nairobi, Kenya; ^3^International Institute of Tropical Agriculture (IITA), Dar es Salaam, Tanzania

**Keywords:** microbial diversity, native microbiome, spore morphological analysis, ecosystem services, sustainable farming

## Abstract

Elucidating the diversity of native arbuscular mycorrhizal (AM) fungi is essential for the sustainable management of semi-arid land ecosystems. This is because they significantly improve plant nutrient uptake and decrease the stress caused by biotic and abiotic factors. In this study, we examined the AM fungal communities and the key drivers influencing their diversity and occurrence in the smallholder farming systems of Eastern Kenya. Soils samples were collected from 34 diverse agricultural fields and AM fungal spores were extracted using wet-sieving and decantation techniques. The spores were quantified, and AM fungal communities were identified based on their morphological characteristics. Statistical data analyses, including relative abundance, the Shannon-Wiener index, analysis of variance (ANOVA), and principal component analysis (PCA), were performed using R software 4.4.0. The results revealed that two AM fungal families dominated the agricultural fields, namely *Gigasporaceae* (61.0%) and *Acaulosporaceae* (39.0%). These fungal families comprised a total of five genera, with the following relative abundances: *Acaulospora* (39.0%), *Gigaspora* (35.05%), *Scutellospora* (23.92%), *Dentiscutata* (1.32%), and *Rococetra* (0.72%). The AM fungal morpho-species were ranked from 1 to 26 across the five genera. *Acaulospora denticulata* ranked the highest, with a proportion of 25.19%. The Shannon-Wiener diversity index revealed a higher diversity of AM fungi in agricultural fields with greater spore richness. The PCA showed that the composition of AM fungal communities was strongly related to soil physiochemical characteristics. Dryland farming systems also played a role in AM fungal composition. Overall, the distribution of AM fungal communities across the agricultural fields was lower, implying the need to adopt sustainable dryland farming systems to enhance native AM fungal communities and support the development of context-specific biofertilizers.

## 1 Introduction

Semi-arid lands (SALs) cover ~40% of the terrestrial land surface and account for ~40 % of global net primary productivity (Wang et al., 2012). In East Africa, over 250 million people depend on SALs for their livelihoods (De Leeuw, 2014). In Kenya, SALs are estimated to occupy 89–90% of the total terrestrial surface area (Amwata et al., 2016). These areas receive erratic rainfall between 300 and 600 mm annually (Vohland and Barry, 2009). SALs are synonymous with ecological degradation, such as soil erosion (Bishaw et al., 2013), which limits agricultural production and rural settlement (AbdelRahman et al., 2023). These regions are continually undergoing massive land use changes due to an increasing population, leading to the transformation of natural ecosystems into crop and animal farmlands (Mganga et al., 2024). Climate change remains a key challenge (Aguilar et al., 2024; Ntinyari and Gweyi-Onyango, 2020), and anthropogenic activities worsen the challenges faced in SALs, making them more severe and unpredictable (Coleine et al., 2024).

SALs host a diverse array of microorganisms that are either free-living or symbiotic (Coleine et al., 2024). One such key microbiome is arbuscular mycorrhizal (AM) fungi, which form mutualistic relationships with more than 80% of terrestrial plant species, ranging from bryophytes to tracheophytes (Smith and Read, 2010). AM fungi are obligate symbionts (Brundrett and Tedersoo, 2018). They create a healthy soil environment (Gupta, 2020) and provides several other benefits, including enhanced nutrient uptake, notably phosphorus and nitrogen (Smith and Read, 2008). This fungi help plants mitigate abiotic stresses, such as drought (Begum et al., 2019), and protect plant roots from soil-borne pathogen attacks (Del Fabbro and Prati, 2014). This mutual association often increases crop yield (Sakha et al., 2019; Buczkowska and Sałata, 2020) and improves the survival rate of vitro-grown plantlets to 100% (Dushimimana et al., 2022). Furthermore, AM fungi contribute indirectly to soil carbon sequestration by promoting soil aggregation (Leifheit et al., 2014). The exudates released by the extraradical hyphae of AM fungi bind soil particles and enhance soil aggregate stability (Wang et al., 2022). Consequently, understanding the dynamics and variables driving changes in AM fungal communities is necessary for applying AM fungi's biological functions in various fields (Yu et al., 2023).

Various environmental factors influence the AM fungal community, and it is clear that the soil environment imposes a strong selection pressure on resident AM fungi (Helgason and Fitter, 2009). Soil chemistry predominantly influences the dynamics of AM fungal spore abundance, root colonization, distribution, and diversity (Song et al., 2019; Šmilauer et al., 2020). The most pronounced soil factor determining the structure of AM fungal communities is pH (Dumbrell et al., 2010; Melo et al., 2017). Other factors include nitrogen (Silvana et al., 2020), phosphorus (Zhao et al., 2017; Ezeokoli et al., 2020), organic matter (Wang et al., 2015), and soil moisture (Veresoglou et al., 2013; Deepika and Kothamasi, 2015). Soil type is also another major factor shaping AM fungal communities. Van Geel et al. (2018) noted that there is a difference between AM fungal communities in acidic and calcareous grasslands. Climate change, particularly increased aridity, can reduce AM fungal diversity and abundance by limiting soil carbon and nitrogen storage due to primary production constraints (Delgado-Baquerizo et al., 2016). The community dynamics of AM fungi also depend on dispersal limitations, abiotic filtering, and biotic interactions (Vályi et al., 2016; Zhang et al., 2021).

One of the principal pillars of agroecology is soil health (HLPE, 2019), which is in line with the Kunming-Montreal Global Biodiversity Framework. This framework aims to ensure that at least 30% of degraded terrestrial land areas are effectively restoredby 2030 (Global Biodiversity Framework, 2022). Therefore, it is fundamental to continuously monitor natural microflora and characterize their exact roles in addressing the degradation of ecosystems and fostering resilience since native microorganisms are effective at colonizing soils and providing ecosystem services, especially in poor soils (Jiang et al., 2023). The main goal of this study was to examine the diversity and structure of native AM fungal communities in the semi-arid lands of Eastern Kenya. Despite their roles in SALs, limited studies have investigated these communities. We hypothesized that (i) the abundance and diversity of AM fungal spores differ between agricultural fields and (ii) AM fungal communities are related to edaphic and climatic factors. We anticipate that our study will support the adoption of appropriate dryland farming systems and the need for agricultural field restoration in SALs to maintain their ecological functions through the rational application of AM fungal inoculum.

## 2 Materials and methods

### 2.1 Study area

**Figure 1:**
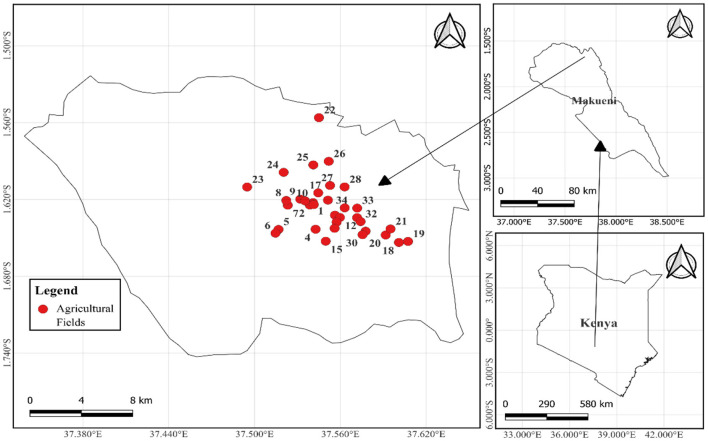
Map of Makueni County showing the location of the sampled agricultural fields. The numbers 1 to 34 marked in red indicate each of the sampled agricultural fields.

This study was conducted in Makueni County, Mbooni sub-county, Eastern Kenya (Figure 1). Makueni is situated at altitudes ranging from 600 m to 1,280 m above sea level (Saiz et al., 2016). The region receives an annual rainfall between 300 and 800 mm, with annual temperatures varying between 20°C and 36°C. Makueni County covers an area of ~8, 035 km^2^ and has a population of 987,550, according to the 2019 National Census (KNBs, 2019). The area is characterized by smallholder mixed farming, with farmers engaging in both livestock and crop farming. The major crops cultivated include maize (*Zea mays*), pigeon peas (*Cajanus cajan*), sorghum (*Sorghum bicolor*), cowpeas (*Vigna unguiculata*), millet (*Eleusine coracana*), common beans (*Phaseolus vulgaris*), green grams (*Vigna radiata*), and mangoes (*Mangifera indica*) (Makueni County Integrated Development Plan, 2023).

### 2.2 Soil sampling and handling

Soil samples, from which AM fungal spores were isolated, were collected from 34 farmer-managed agricultural fields (Figure 1), representing a diversity of farms in this Agroecological Living Landscapes (ALLs). The owners were selected during a co-design workshop implemented by a team of experts from the Agroecology Initiative of Consultative Group on International Agricultural Research (CGIAR) (Fuchs et al., 2023). The selected fields were primarily used for growing a variety of crops, including maize, common beans, cowpeas, pigeon peas, and green grams. Soil amendments used by the farmers included organic fertilizers, inorganic fertilizers, or a combination of the two (Table 1). Baseline soil samples were collected during the dry season of late September 2023 when root activities had declined and AM fungal spores were expected to be highly sporulating (Jefwa et al., 2006).

**Table 1:** GPS location, cropping system, and types of soil amendments in the agricultural fields where soil samples for AM fungal spore isolation were collected.

**Field no**.	**Latitude**	**Longitude**	**Type of crops**	**Soil amendments**
1	−1.62245	37.54090	Maize, Common beans, Cowpeas	None
2	−1.62429	37.53861	Maize, Common beans, Pigeon peas, Sunflower	Farmyard, inorganic
3	−1.62401	37.54139	Maize, Common beans, Pigeon peas	Compost
4	−1.64321	37.54268	Maize, Cowpeas, Pigeon peas	Inorganic
5	−1.64337	37.51675	Maize, Common beans, Pigeon peas	Farmyard
6	−1.64629	37.51465	Maize, Common beans, Cowpeas, Pigeon peas	Farmyard, inorganic
7	−1.62443	37.52322	Maize, Common beans, Pigeon peas	Farmyard, inorganic
8	−1.62075	37.52216	Maize, Common beans	Farmyard
9	−1.61974	37.53195	Maize, Common beans	Inorganic
10	−1.62105	37.53531	Beans	Inorganic
11	−1.63223	37.55619	Maize, Common beans	Compost
12	−1.63401	37.55970	Maize, Common beans, Pigeon peas	Farmyard
13	−1.63766	37.55737	Maize, Common beans, Pigeon peas	Farmyard, Compost
14	−1.64249	37.55597	Maize, Common beans, Pigeon peas	Farmyard, inorganic
15	−1.65256	37.54976	Maize, Common beans	Farmyard
16	−1.62049	37.55130	Maize, Common beans	Farmyard
17	−1.61483	37.54450	Maize, Common beans, Cowpeas	Farmyard
18	−1.65352	37.60094	Maize, Common beans, Pigeon peas	Farmyard
19	−1.65273	37.60730	Maize, Common beans, Cowpeas, Pigeon peas	Farmyard, inorganic
20	−1.64771	37.59169	Maize, Common beans, Cowpeas, Pigeon peas	Farmyard
21	−1.64299	37.59505	Maize, Common beans, Cowpeas, Pigeon peas	Farmyard
22	−1.55612	37.54502	Maize, Common beans, Cowpeas, Pigeon peas	Farmyard
23	−1.61025	37.49482	Maize, Common beans, Pigeon peas	Farmyard, inorganic
24	−1.59880	37.52033	Maize	None
25	−1.59300	37.54108	Maize, Common beans, Cowpeas	Farmyard, inorganic
26	−1.59019	37.55186	Maize, Common beans, Pigeon peas	Farmyard
27	−1.60905	37.55277	Maize, Common beans, Cowpeas	Farmyard
28	−1.61016	37.56296	Maize, Common beans, Green grams	Farmyard
29	−1.64473	37.57764	Maize, Common beans	Farmyard
30	−1.64745	37.57552	Maize	Farmyard
31	−1.63420	37.57171	Maize, Common beans	None
32	−1.63726	37.57414	Maize, Common beans	Farmyard
33	−1.62672	37.57176	Maize, Common beans	Farmyard
34	−1.62650	37.56304	Maize, Common beans, Green grams	Farmyard

**Figure 2:**
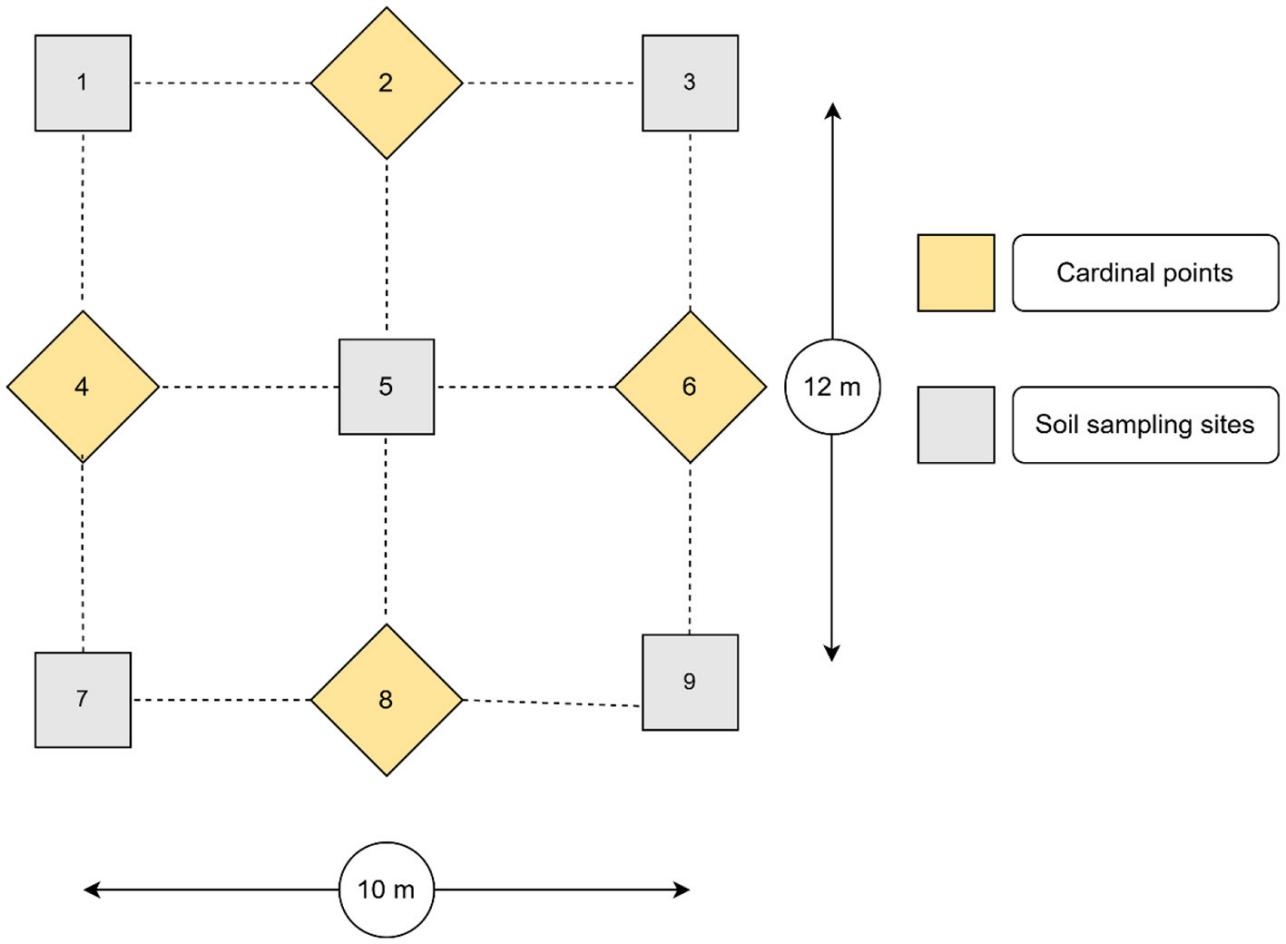
Overall soil sampling scheme for AM fungal spore isolation from a plot. The method was adapted from the Society for the Protection of Underground Networks (2023). The numbers 1, 3, 5, 7, and 9 represent the soil sampling points, while the numbers 2, 4, 6, and 8 represent the cardinal directions.

One soil sampling plot, measuring 12 m by 10 m, was delineated in each of the selected agricultural fields. Thereafter, nine sub-samples (5 cm in diameter and 20 cm in depth) were collected from each plot, following the adapted steps described in Figure 2 (Society for the Protection of Underground Networks, 2023). After removing any loose litter, the soil sampling procedure proceeded by collecting the first sample from the central point (number 5). In addition, four sampling points were established, with 3 m measured in the cardinal directions 2 and 8 from the central point and 2.5 m measured in the cardinal directions 4 and 6 from the central point. A sample was collected at each of these points, resulting in a total of four samples. Furthermore, from the projected four corners (1, 3, 7, and 9), four soil samples were collected. The soil sub-samples from each plot were manually homogenized by mixing them in a clean bucket. A 1 kg sample was drawn from the composite sample, stored in a plastic bag, and labeled with the owner's name, GPS coordinates, field history, and sampling date. Finally, 34 soil samples were collected.

### 2.3 Spore isolation and morphological characterization

The collected soils were wet-sieved and sucrose-centrifuged to extract AM fungal spores, following the method described by Gerdemann and Nicolson (1963) with modifications made by Ingleby (2007). In summary, 50 g of rhizosphere soil was suspended in 500 mL tap water and stirred for 1 min. The solutions were then sequentially sieved through mesh sizes of 710, 400, 200, 100, and 45μm under flowing tap water to separate the spores by size. The soil fraction from each sieve was collected into a beaker. Then, the spore suspensions were transferred to 50 mL centrifugation tubes and centrifuged with a sucrose-water solution (20% and 60% w/v) for 5 min at 2,700 rpm (Sakha et al., 2024). The supernatant was decanted into a 45-μm sieve, washed, and transferred to Petri dishes for quantification under a stereomicroscope. The spores were enumerated and distinguished according to their morphological characteristics (such as spore size, color, surface appearance, and hyphal attachment).

Some spore morphotypes were mounted on slides in polyvinyl–lactic acid–glycerine (PVLG) and a mixture of PVLG with Melzer's reagent (1:1; v/v) (Morton, 1991) to observe wall structures and other specific attributes using a compound microscope at 40 × magnification (Zeiss standard microscope). Then, the spores were identified to the species level or as a specific morphotype using descriptions and identification criteria based on the method described by Schenck and Perez-Collins (1990), online references of species descriptions from the International Culture Collection of Vesicular–Arbuscular Mycorrhizal Fungi website (INVAM) (http://fungi.invam.wvu.edu/the-fungi/species-descriptions.html), and descriptions in the literature.

### 2.4 Diversity measures

#### 2.4.1 Shannon-Wiener diversity index

The Shannon-Wiener index is a method used to measure the diversity of species in a community. This index is calculated by taking the ratio of the number of species to their importance values (e.g., biomass or productivity) within a trophic level or community, as indicated in the equation below:


$$H\prime =\;\sum_{l=i}^{s}\left(\frac{ni}{N}\;\log_{2}-\frac{ni}{N}\right)$$

Where *H'* is the Shannon-Wiener diversity index, s is the number of species in a sample, *N* is the total number of species, and *ni* is the number of individuals within a species. The higher the value of *H'*, the higher the diversity of species in a particular community. A value of *H'* = 0 indicates a community with only one species.

#### 2.4.2 Spore abundance, relative abundance, and species richness

The spores were counted in three replicates under a stereomicroscope at 40 × magnification, and spore abundance was expressed as the number of spores per 50 g of soil.

Relative abundance (RA %) was estimated using the formula: (number of spores of individuals of a given species)/(the total number of individuals in a community) × 100 (Kumar and Ghose, 2008).

Species richness was measured as the number of AM fungal species present in the soil sample.

### 2.5 Soil analysis

The samples were scanned using a Bruker Tracer 5i Portable X-ray Fluorescence (pXRF) instrument to determine the total elemental concentrations of magnesium (Mg), aluminum (Al), phosphorous (P), sulfur (S), potassium (K), calcium (Ca), manganese (Mn), iron (Fe), copper (Cu), and zinc (Zn). The samples were predicted using the ICRAF's global soil models. The models were fitted using the Bayesian regularization for feed-forward neural networks (BRNN) and random forest (RF) algorithms. The performance of the model was evaluated using a 30% hold-out validation set. The predicted soil properties included soil pH, exchangeable Al, Ca, Cu, Fe, K, Mg, Mn, CEC, and S, phosphorus sorption index (PSI), soil organic carbon (SOC), and total nitrogen (TN). The soil pH was determined using a pH meter at a soil-to-water ratio of 1:2.5 (w/v) (Bao, 2000). Exchangeable Al, Ca, Cu, Fe, K, Mg, Mn, CEC, and S were determined using the Mehlich 3 extraction method (Mehlich, 1984). The phosphorus sorption index (PSI) was calculated based on the molar ratio of P to Al or Al plus Fe (Dari et al., 2015). Soil organic carbon (SOC) was determined using K_2_Cr_2_O_7_ digestion (Walkley and Black, 1934), and total nitrogen (TN) was analyzed using both Kjeldahl and HClO_4_-H_2_SO_4_ methods (Yang et al., 2023).

### 2.6 Statistical analysis

All data were checked for normality and homogeneity of variance, and the data with a non-normal distribution were log (x + 1) transformed to ensure a normal distribution. Spore relative abundance and the Shannon-Wiener index of diversity (H) were calculated. To understand the relationship between AM fungal community structure and soil physicochemical parameters, principal component analysis (PCA) was performed. To compare AMF diversity between agricultural fields, a one-way analysis of variance (ANOVA) was performed, and Tukey's test was used to analyze the differences in spore abundance at a *p*-value of < 0.05 using R software (version 4.4.0).

## 3 Results

### 3.1 Presence of native arbuscular mycorrhizal fungi in cultivated soils

**Figure 3:**
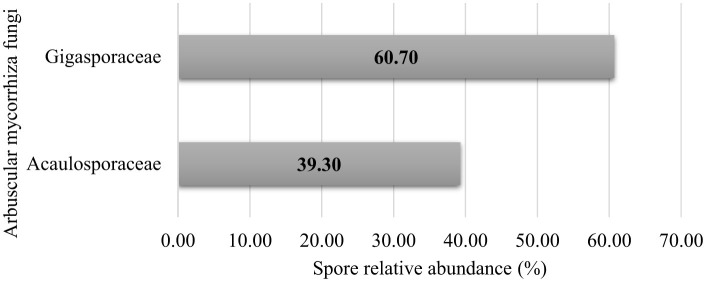
Family-wise taxonomic profile composition of native arbuscular mycorrhizal fungi communities in cultivated soils of Makueni County.

The study revealed the presence of two native AM fungal families, distinguished based on spore morphological features such as spore color, spore size, spore ornamentation, spore shape, and hyphal attachments on the spore (INVAM and Schenck and Perez-Collins, 1990). The relative abundances of the two families isolated from the rhizosphere soil primarily revealed the dominance of *Gigasporaceae* and *Acaulosporaceae* (Figure 3).

**Figure 4:**
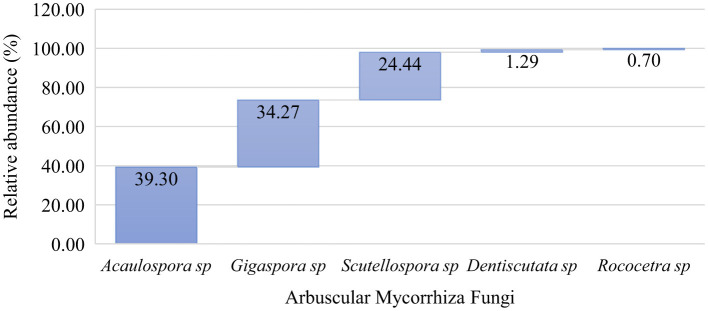
Genus-wise taxonomic profile composition of native arbuscular mycorrhizal fungal communities in the cultivated soils of Makueni County.

Based on the morphological appearances, five genera from the sub-family *Glomeromycota* were identified (Figure 4). The analysis of spore relative abundance showed that the most common genera, in order of superiority, were *Acaulospora, Gigaspora, Scutellospora, Dentiscutata*, and *Rococetra*. Of the two AM fungal families (Figure 4), *Gigasporaceae* consisted of two genera, namely *Gigaspora* and *Scutellospora*, while *Acaulosporaceae* comprised three genera, namely *Acaulospora, Dentiscutata*, and *Rococetra*.

The AM fungal morpho-species isolated from the SAL farming systems exhibited variations in spore abundance per 200 g of soil. A total of 26 morpho-species were isolated (Table 2). While all morpho-species were recorded in the area, they differed significantly in spore abundance, with the highest count being 198 spores and the lowest being 1 spore per 200 g of soil. However, this is very low since it was a cumulative measure from the 34 agricultural fields. The analysis of spore abundance indicated that *Acaulospora denticulata* had the highest rank, accounting for a proportion of 24.57%. Based on the rankings, three species—*Acaulospora denticulata, Gigaspora margarita*, and *Scutellospora* sp. 5—recorded the largest proportion, accounting for ~54.09%, which is slightly higher than half of the total proportion. In addition, nine spores could not be identified at the species level because they lacked enough distinct features.

**Table 2:** Rank of the total number of arbuscular mycorrhizal fungal spores per species and their proportions in 200 g^−1^ of air-dried soil.

**Morpho-species**	**Rank**	**Total number of spores**	**Proportion (%)**
*Acaulospora denticulata*	1	198	24.57
*Gigaspora margarita*	2	156	19.35
*Scutellospora sp 5*	3	82	10.17
*Scutellospora sp 1*	4	50	6.20
*Gigaspora albida*	5	42	5.21
*Gigaspora sp 1*	6	39	4.84
*Acaulospora koskei*	7	32	3.97
*Scutellospora sp 2*	8	31	3.85
*Acaulospora sp 1*	13	25	3.10
*Scutellospora sp 4*	9	22	2.73
*Acaulospora laevis*	10	19	2.36
*Acaurospora delicata*	11	17	2.11
*Gigaspora sp 2*	12	15	1.86
*Acaulospora colombiana*	14	15	1.86
*Scutellospora sp 3*	15	12	1.49
*Scutellospora scutata*	16	8	0.99
*Dentiscutata heterograma*	17	8	0.99
*Gigaspora rosea*	18	7	0.87
*Racocetra castanea*	19	6	0.74
*Acaulospora rehmii*	20	5	0.62
*Acaulospora tuberculata*	21	4	0.50
*Scutellospora calospora*	22	4	0.50
*Dentiscutata nigra*	23	3	0.37
*Acaulospora sp 2*	24	3	0.37
*Gigaspora decipiens*	25	2	0.25
*Scutellospora biornata*	26	1	0.12

### 3.2 Genus-wise taxonomic profile composition of the native arbuscular mycorrhizal fungal communities in the study fields

**Figure 5:**
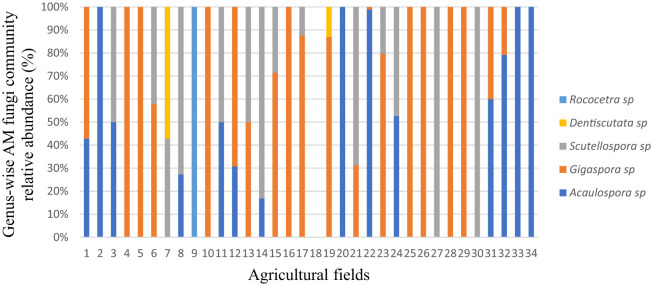
Genus-wise taxonomic profile composition of native arbuscular mycorrhizal fungal communities' relative abundance in different agricultural fields.

The genus-wise taxonomic profile showed that *Gigaspora* sp. dominated in 20 agricultural fields (Figure 5). Furthermore, eight fields had 100% *Gigaspora* sp., and four had 100% *Acaulopora sp*. Two fields had 100% *Scutellopora sp*, two fields each recorded 100% *Rococetra* sp., and four fields recorded 100% *Dentiscutata* sp., *Acaulospora* sp., and *Scutellospora* sp. or *Gigaspora* sp. *Scutellospora* sp., together with *Gigaspora* sp., occurred in five fields. In addition, *Dentiscutata* sp., along with *Scutellospora* sp. or *Gigaspora* sp., was isolated in one field each. However, only one field did not record any AM fungal species. Overall, *Gigaspora* sp. was found in 20 fields, followed by *Acaulospora* sp., and *Scutellospora* sp., which was found in 14 fields each.

### 3.3 Arbuscular mycorrhizal fungal diversity indices and spore abundance in agricultural fields

Approximately 18 fields recorded a spore richness of 2, while the remaining fields had a spore richness of 1, where AM fungi were present (Table 3). The Shannon-Wiener diversity index revealed that AM fungal diversity was higher in the fields with greater spore richness, with only six fields having an approximate index of 0.6. Spore abundance was significantly different between the fields (Table 3). The average number of AM fungal spores ranged from 0 to 61.30 per 50 g of dry soil^−1^ in the area. The highest spore abundance, 61 spores, was recorded in field number 22, followed by 34 spores in field number 14 (Table 3). The remarkable difference in the AM fungal spore abundance and communities observed within the agricultural fields could be due to several factors, including the types of cropping systems and soil amendments (Table 1). The field that exhibited high AM fungal spore abundance previously had maize, beans, cowpeas, and pigeon peas as intercrops, with farmyard manure as a soil amendment. This demonstrates that dryland farming systems affect AM fungal spore abundance and composition, mainly due to the interactions between plant species.

**Table 3:** Arbuscular mycorrhizal fungal diversity indices in different agricultural fields.

**Fields no**.	**Spore richness**	**Shannon-Wiener index**	**Spore abundance**
1	2.00	0.68	4.00 ± 1.00^klm^
2	1.00	0.00	5.00 ± 1.00^ijkl^
3	2.00	0.69	2.67 ± 0.57^mno^
4	1.00	0.00	14.7 ± 0.57^e^
5	1.00	0.00	7.67 ± 0.57^gh^
6	2.00	0.00	5.67 ± 0.57^hijk^
7	2.00	0.68	5.00 ± 1.00^ijkl^
8	2.00	0.58	17.0 ± 1.00^d^
9	1.00	0.00	2.67 ± 0.57^mno^
10	1.00	0.00	4.33 ± 0.57^klm^
11	2.00	0.69	13.7 ± 0.57^e^
12	2.00	0.61	9.67 ± 0.57^fg^
13	2.00	0.69	3.67 ± 0.57^klmn^
14	2.00	0.45	34.0 ± 1.00^b^
15	2.00	0.59	10.7 ± 0.57^f^
16	1.00	0.00	1.67 ± 0.57^nop^
17	2.00	0.37	3.67 ± 0.57^klmn^
18	0.00	0.00	0.00 ± 0.00^p^
19	2.00	0.38	9.67 ± 0.57^fg^
20	1.00	0.00	3.33 ± 0.57^lmn^
21	2.00	0.62	4.67 ± 0.57^jklm^
22	2.00	0.62	61.3 ± 1.53^a^
23	2.00	0.50	21.0 ± 1.00^c^
24	2.00	0.38	9.67 ± 0.57^fg^
25	1.00	0.00	7.00 ± 1.00^hi^
26	1.00	0.00	4.33 ± 0.57^klm^
27	1.00	0.00	6.67 ± 0.57^hij^
28	1.00	0.00	4.67 ± 0.57^jklm^
29	1.00	0.00	1.00 ± 0.00°*p*
30	1.00	0.00	1.00 ± 0.00°*p*
31	2.00	0.67	6.67 ± 0.57^hij^
32	2.00	0.51	9.67 ± 0.57^fg^
33	1.00	0.00	1.67 ± 0.57^nop^
34	1.00	0.00	9.67 ± 0.57^fg^
*P*-value			< 0.001

Mean values followed by the same letter within a column are not statistically significant at a *p*-value of < 0.001 between the agricultural fields.

### 3.4 The relationship between AMF spore abundance, elevation, and latitude

**Figure 6:**
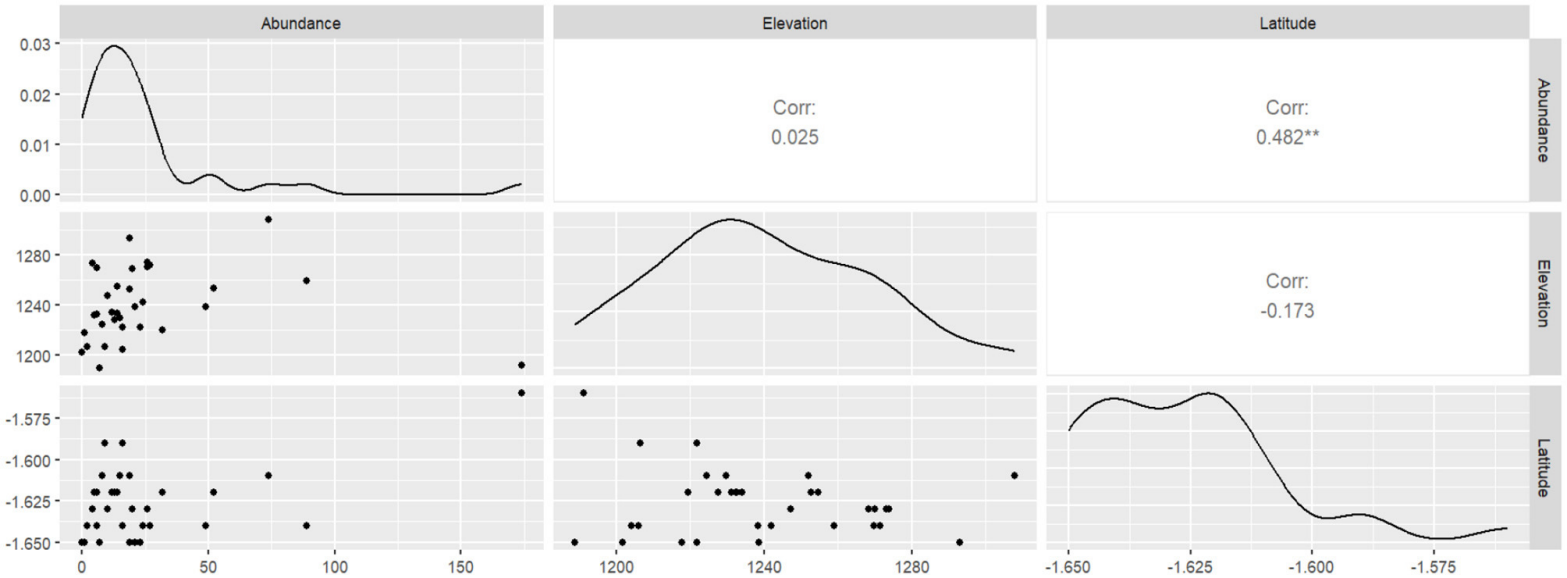
Pearson correlation analysis between arbuscular mycorrhiza fungal abundance and geographic coordinates.

We found that AM fungal spore abundance had a moderate positive correlation with latitude (0.482^**^), although it had a very weak positive correlation of 0.025 with elevation (Figure 6).

### 3.5 The relationship between the AMF spore abundance, genera, and soil physicochemical properties

**Figure 7:**
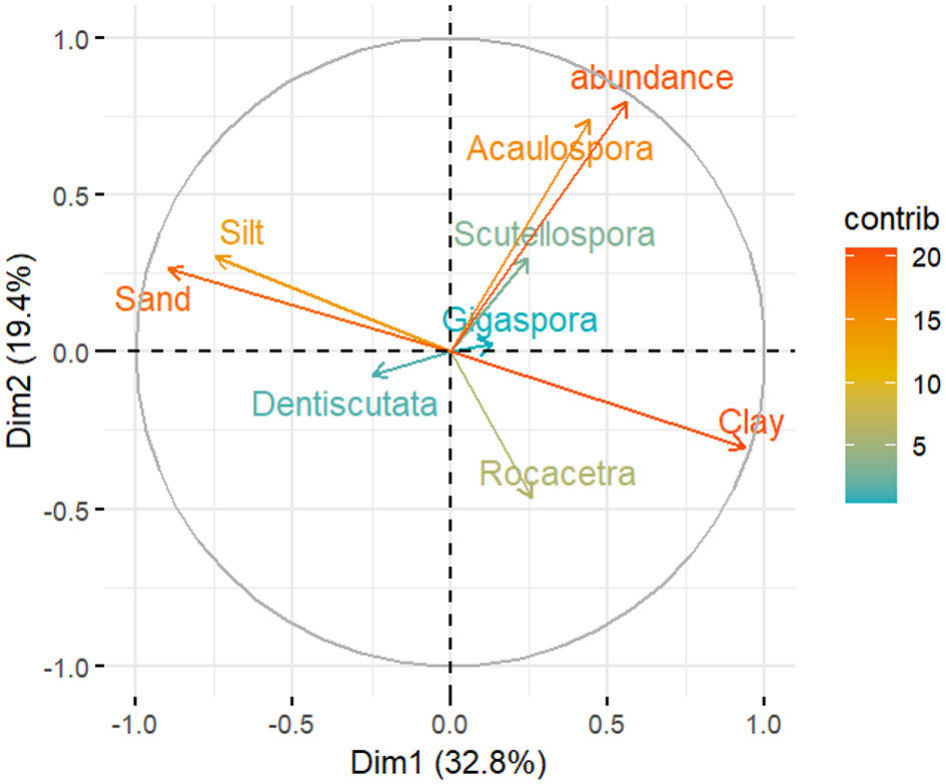
Principal component analysis (PCA) of AM fungal spore abundance, AM fungal communities, and soil physical properties across the 34 sampling sites in the dryland farming system (with the mean values). The correlation circle (correlation = 1) shows the relationship between the variables and dimensions (Dim-1 and Dim-2). Variable points away from the origin are well represented on the factor map.

**Figure 8:**
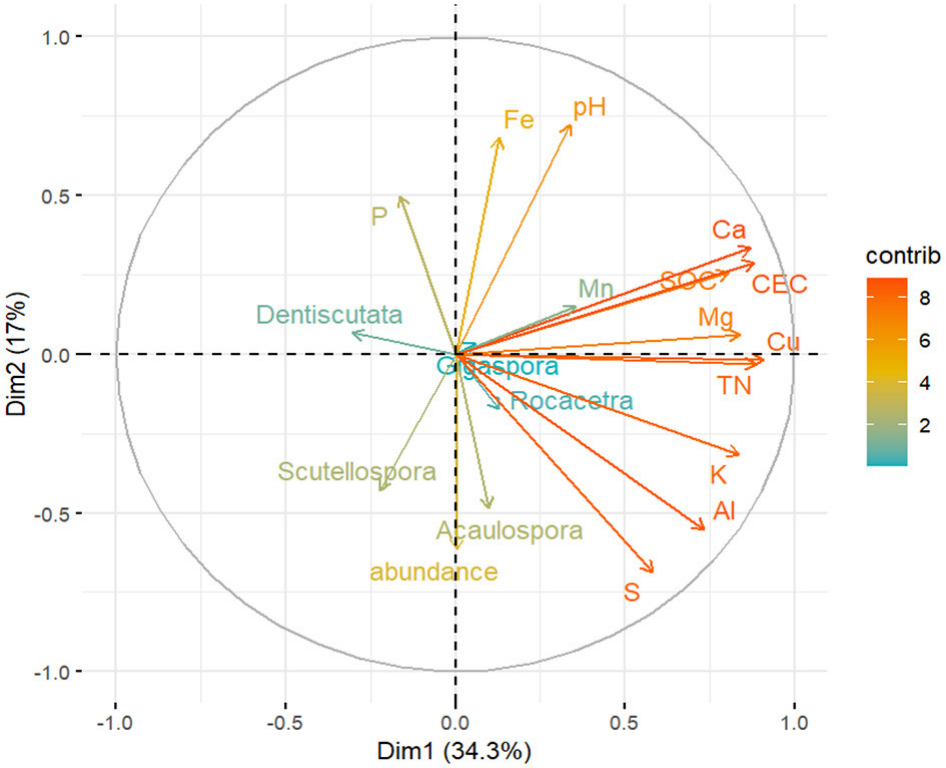
Principle component analysis (PCA) of the AM fungal spore abundance, AM fungal community genera, and soil chemical properties across the 34 sampling sites in the dryland farming system (with the mean values). The correlation circle (correlation = 1) shows the relationship between the variables and dimensions (Dim-1 and Dim-2). Variable points away from the origin are well represented on the factor map.

The correlation between the AM fungal community structure and soil properties is presented in Figures 7, 8. In the PCA plots, the length of the arrows represents the relative importance affecting the community, while the angle between the variables indicates the degree to which factors are correlated, with a smaller angle reflecting a higher correlation. The resulting PCA analysis of the soil physical properties, AMF spore abundance, and AM fungal communities is presented in Figure 7. The first two latent variables (Dim-1 and Dim-2) accounted for more than 52% of the variation in AM fungal genera and soil physical properties. The total spore abundance, as well as the sand and clay contents, showed very high variance, whereas the spore abundance of the genera *Gigaspora* and *Dentiscutata* showed low variance in relation to sand and clay contents. The spore abundance and AM fungal genera showed a weak correlation with clay, sand, and silt.

The PCA of soil chemical properties revealed that S, Al, K, Cu, TN, SOC, CEC, and Ca were the key factors that shaped the AM fungal community (Figure 8). The first two latent variables, Dim-1 and Dim-2, accounted for 34.3% and 17% of the variability, respectively. The spore abundance of AM fungi and the genus *Acaulospora* were positively correlated with S, Al, K, Cu, and TN. However, these elements negatively influenced the genera *Dentiscutata* and *Gigaspora*. Spore abundance, *Acaulospora*, and *Scutellospora* were negatively correlated with P, Fe, and pH.

**Figure 9:**
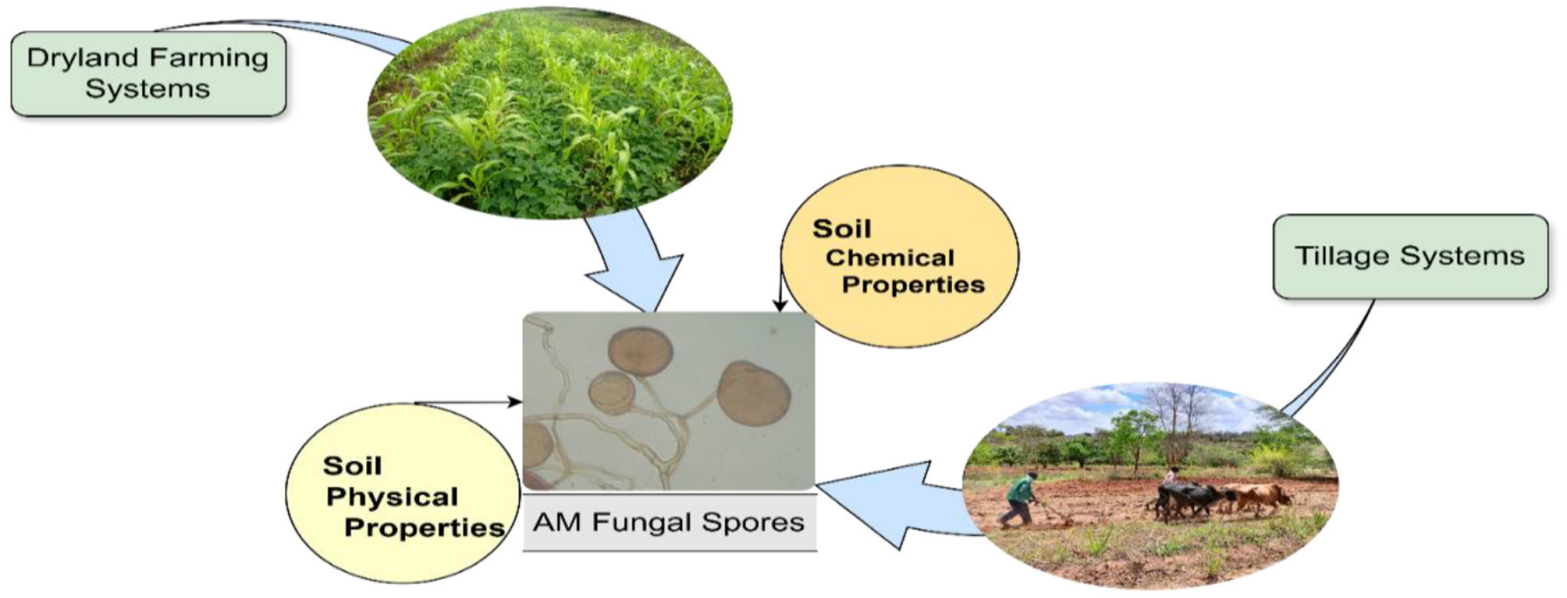
Conceptual representation of factors that affect arbuscular mycorrhizal fungal community structure at a finer spatial scale in drylands farming systems. The arrows with greater thickness indicate the main above-ground agricultural management practices, such as cropping systems and tillage systems, while the thin arrows indicate the below-ground soil properties, such as soil physiochemical properties, that influence AM fungal community structure in an agricultural field.

The results of the redundancy analysis are presented using the spore abundances of the different AM fungal genera. Integrating the above results, a comprehensive model was established to identify the factors that jointly affect the distribution of AM fungal communities at a finer spatial scale in dryland agroecosystems (Figure 9).

## 4 Discussion

Few studies have explored AM fungal communities and their drivers in low-input dryland farming systems. This study, therefore, is a significant contribution to our understanding of these organisms' ecological preferences at each site. It is recommended that morphological and molecular characteristics be combined for AM fungal identification at the species level because both methods provide unique and crucial information (Overby et al., 2015; Säle et al., 2015). Ideally, both methods have their respective advantages and limitations (Oehl et al., 2017). This study used the morphological characterization due to some limitations, although we banked on its advantages and the fact that it can provide evidence unaffected by potential biases introduced during PCR steps. However, its limitations include challenges in identifying cryptic species or the underrepresentation of non-sporulating taxa. Based on the most reliable morphological features and through direct AM fungal spore isolation from the rhizosphere soil, two AM fungal families, *Gigasporaceae* and *Acaulosporaceae*, were unexpectedly distinguished. This denotes that these two are the most dominant families associated with low-input SAL farming systems. Egerton-Warburton et al. (2007) observed a higher abundance of *Gigasporaceae* and *Acaulosporaceae* in typically mesic environments than in semiarid ecosystems, although no such comparison was performed in the present study.

SALs host many endemic species, including AM fungi, which exhibit unique adaptations to harsh dryland conditions (Kamalvanshi et al., 2012). Currently, the primary driving factor of AM fungal communities is habitat conditions (Vasar et al., 2022), and these AM fungal communities respond to these conditions with lower diversity and less variability. The distribution of AM fungal genera occurred in patches, with some restrictions to specific agricultural fields, probably due to habitat filtering. This implies a finer-scale spatial selection of AM fungi species, particularly by tillage practices, cropping systems, and edaphic factors. Our findings suggested that the AM fungal communities in the soil responded spatially to the SAL farming systems. The main cropping system within the agricultural field was intercropping, but the intercrops varied from one agricultural field to another. In addition, different soil amendments were used for growing the crops. This provided clear evidence that SAL farming systems affected the AM fungal spore abundance and composition mainly due to the interactions between plant species. Our results are consistent with those of Yang et al. (2012), who observed that AM fungal community composition was significantly correlated with the plant community among the ecosystems. Similarly, Egbokaa et al. (2022) concluded that the cropping system significantly influenced the population density of AM fungi. For instance, monocropping records lower AM fungal diversity due to the limited diversity of hosts (Burrows and Pfleger, 2002; Oehl et al., 2003). Moreover, Liu et al. (2012) demonstrated that the types of host plant species grown have a strong and significant impact on AM fungal diversity and distribution. Furthermore, Säle et al. (2015) reported that the type of fertilization significantly affects AM fungal spore populations.

Among all the AM fungi species, the *Gigasporaceae* family cumulatively had more species than *Acaulosporaceae*. Members of the *Gigasporaceae* family are known for their competitive life strategy, characterized by increased hyphal production, higher carbon demand, and enhanced phosphorus absorption from the soil. These traits are important in environments with low levels of soil nutrients (Maherali and Klironomos, 2007; Chagnon et al., 2013). Notably, Hart and Reader (2002) revealed that this family produces more extraradical mycelia in the soil than intraradical mycelia within root systems. Mycelia provide improved opportunities for soil exploration, ensuring a better nutritional contribution to the hosts. In addition, through increased mycelial production, the species in the family may help stabilize the growth media and contribute to increased soil aggregation (Vieira et al., 2020).

The family *Gigasporaceae* consisted of two genera, *Gigaspora* and *Scutellospora*, while *Acaulosporaceae* comprised three genera, namely *Acaulospora, Dentiscutata*, and *Rococetra*. The genus *Acaulospora* showed a high relative spore abundance and was more frequent (Figures 3, 4). Studies have shown that this genus sporulates more rapidly than *Scutellospor*a and *Gigaspora* in the same environment (Jefwa et al., 2012; Oehl et al., 2009). At the same time, our findings closely align with those of Cuenca et al. (1998) and Boddington and Dodd (2000), who detailed the superiority of *Acaulospora* species in disturbed soils, one of the characteristics of the agricultural fields in the study area. This observation aligns with those of De Pontes et al. (2024), who reported the predominance of *Acaulospora* species in dry soils of the Brazilian Cerrado, transitional areas toward the Caatinga, and the Atlantic Forest, possibly due to their adaptation to moisture conditions. Accordingly, Xavier Martins and Rodrigues (2020) revealed that *Acaulospora* species are associated with low-input farming systems and forest and grassland soils. *Acaulospora* species are considered facultative symbionts adapted to a broad range of soils and host species (Sieverding et al., 1991; Shepherd et al., 1996; Straker et al., 2010).

The diversity indices and the spore abundance of AM fungi were lower and varied significantly across the agricultural fields, with each field presenting one or two genera (Table 3). This could be due to the farming systems. Boddington and Dodd (2000) showed that soil disturbance caused by agricultural activities directly affects AM fungal propagule availability as richness and spore density are reduced, along with the length of AM fungal extraradical mycelium. Furthermore, the AM fungal diversity index showed a trend toward the dominance of AM fungal diversity in fields with high spore richness; however, this did not necessarily result in higher spore abundance. The diversity reported in the current study was particularly low, considering that soil sampling was performed at a single point in time. Sampling other land use types beyond cropland or across different seasons could have likely revealed greater spore diversity and density, as spores represent the dormant stage of AM fungi. Their production in natural systems varies seasonally (Bever et al., 2001), which likely impacted the number of species detected in our study. Inadequate exploration of seasonal variation in AM fungal spore production and diversity has been conducted in drylands with biannual precipitation patterns. We collected soils samples in September during the dry season, and as a result, certain AM fungal taxa were not captured because they may preferentially sporulate during the wet season or do not sporulate at all.

Elevation lays the foundation for investigating the driving forces that lead to species aggregation since different elevation gradients provide a unique opportunity to explore AM fungal community distribution and diversity (Wagg et al., 2011). In this study, we found that AM fungal abundance had a weak positive correlation with elevation, while spore abundance showed a moderate positive correlation with latitude (Figure 6). This could be due to the fact that the sampled agricultural fields were located within the same elevation and latitude zones, thereby limiting the influence of these factors on spore diversity and abundance. The finding is consistent with those of landscape-scale studies by Hazard et al. (2013), Xiang et al. (2014), and De Beenhouwer et al. (2015), which revealed that AM fungal communities are primarily structured by local abiotic conditions rather than geographic distance. This finding is an indicator that elevation and latitude play a moderate role in determining the assembly of contemporary AM fungal communities at a local level.

Our study provides direct evidence of environmental filtering by edaphic factors in SAL farming systems. The results showed that spore abundance and AM fungal communities were highly influenced by soil textural characteristics, such as clay, sand, and silt, although their correlation was weak (Figure 7). This could be because of the general composition of soil textural classes in the present study; for instance, loam soil was not detected in the agricultural fields. In support of this, soil texture has been identified as a factor that affects mycorrhizal efficiency (Joshi and Singh, 1995) such as root colonization (Carrenho et al., 2007) and AM fungal community differences (Moebius-Clune et al., 2013). Furthermore, evidence indicates that AM fungal community composition differs greatly between soil types in the field. The genus *Acaulospora* showed a slight positive correlation with sand (Mukhongo et al., 2023), the family *Gigasporaceae* was more abundant in sandy soil, and the family Glomeraceae was more abundant in clay soil (Lekberg et al. (2007). These findings were observed under conditions where confounding factors, such as host plants, were minimized. Since the family *Gigasporaceae* was more abundant in the region, it may have had a competitive advantage in the sand.

Regarding soil chemical properties, the results of this study indicated that the AM fungal genera differed in their sensitivity to these properties, with some species being specifically associated with certain elements. Indeed, previous studies have demonstrated the influence of soil chemical properties on the composition of AM fungal communities (Jansa et al., 2014; Xiang et al., 2016). The PCA revealed that the genus *Acaulospora* was closely associated with S, Al, K, and Cu. Soil P, pH, and Fe were significantly and negatively correlated with spore abundance and the genera *Acaulospora* and *Scutellospora*. In contrast, the genus *Dentiscutata* appeared to be more tolerant (Figure 8). Changes in the AM fungal community composition have been examined in relation to P. Notably, Amballa and Bhumi (2011) and Johnson et al. (2015) concluded that available soil P suppressed AM fungi root colonization and spore density. Specifically, Qin et al. (2020) observed that high soil fertility, especially available soil P status, reduced AM fungal development in intensively managed agricultural systems. In this context, Johnson et al. (2013) reported that high concentrations of P were associated with lower diversity of AMF due to optimal resource allocation and biotic interactions. Accordingly, Gomes et al. (2018) revealed a high abundance of *Glomeromycota* when the P content was low, with a prevalence of the genera *Gigaspora, Scutellospora*, and *Rococetra* in the root samples of maize. Zhang et al. (2022) highlighted the possibility of pH affecting the formation and development of AM fungal spores. According to Davison et al. (2021), soil pH is one of the most important abiotic factors regulating microbial communities, particularly AM fungi (Jansa et al., 2014; Da Silva et al., 2014). In this study, we observed specifically that the family *Acaulosporaceae* was among the leading families, and this corroborates with Chagnon et al. (2013) and Li et al. (2020), who reported the presence of this family in environments with low pH. Mukhongo et al. (2023) suggested that the favorable soil pH range supporting AM fungal spore sporulation is from 5.85 to 6.64, Dobo et al. (2016) reported a range of 6.18 to 6.28, and Ketchiemo et al. (2024) reported a range of 6.33 to 6.67—values that are close to neutral. In the current study, the pH value ranged from 5.84 to 7.36. This evidence shows that AM fungi species vary in their response to soil pH, not only at the local scale but also at the landscape level. Since the genus Acaulospora was strongly negatively correlated with soil pH (Figure 8) compared to other genera, this may indicate the coexistence of acid-tolerant and acid-sensitive fungi within the SAL ecosystem. For instance, the family *Acaulosporaceae* is reported to be tolerant of acidic tropical soils (Bagyaraj, 2014; Temegne et al., 2017), and low pH levels (Melo et al., 2019). Other studies have found Fe among the distinct soil chemical factors that influence AM fungi negatively (Vieira et al., 2018; Rodríguez-Rodríguez et al., 2021).

Finally, of the 26 AM fungi species reported in the current study, two species are considered rare (species that occurred in one or two farms). Assis et al. (2014) noted that natural ecosystems contain a higher number of rare species compared to agricultural ones and observed that some species may have disappeared in cultivated soils. Similarly, De Carvalho et al. (2012) found high species richness (49 species) in plant species-rich areas with high plant endemism in rocky fields within the Cerrado savanna, indicating that natural vegetation sites in tropical regions generally support high AM fungal diversity. According to Pagano et al. (2012), semi-arid regions have favorable conditions for the growth of AM fungi. The low diversity of species in agricultural fields in the present study underscores the importance of adopting biodiversity-friendly farming practices. One potential solution is the use of microbial inoculants, particularly AM fungi, which can serve as an eco-friendly alternative to traditional fertilizers and pesticides (Ghorui et al., 2025). Accordingly, Martínez et al. (2024) identified that historical legacies significantly influence the persistence of AM fungi in the field by preserving natural habitats that act as safe place for vulnerable species. Therefore, maintaining natural habitats around agricultural fields can further promote soil AM fungal diversity for future generations.

## 5 Conclusion

Our findings revealed the sensitivity of AM fungal community structure to agricultural farming practices and environmental factors in drylands. This community sensitivity has a substantial impact on their function, which subsequently affects their ecological functions. It was also evident that the spore abundance was lower across all farms, implying the need to adopt sustainable biodiversity-friendly farming practices such as reducing soil disturbance, planting a diverse range of species, avoiding the overuse of fertilizers and chemicals, and incorporating organic matter to support and intensify native AM fungi in the soil. Furthermore, the results prompt a discussion regarding the importance of developing context-specific, highly efficient native AM fungal strains that account for natural adaptations in dryland farming systems. Future research should expand the scope to explore AM fungal dynamics across an entire landscape to achieve vibrant, diverse, and healthy landscapes.

## Data Availability

The raw data supporting the conclusions of this article will be made available by the authors, without undue reservation.
